# Entrainment of Voluntary Movement to Undetected Auditory Regularities

**DOI:** 10.1038/s41598-017-15126-w

**Published:** 2017-11-01

**Authors:** Aaron Schurger, Nathan Faivre, Leila Cammoun, Bianca Trovó, Olaf Blanke

**Affiliations:** 1 Laboratory of Cognitive Neuroscience, Brain Mind Institute, Faculty of Life Sciences, Swiss Federal Institute of Technology (EPFL), Geneva, Switzerland; 2 Defitech Chair in Brain-Machine Interfaces, Center for Neuroprosthetics, Swiss Federal Institute of Technology, Geneva, Switzerland; 3 Center for Neuroprosthetics, Faculty of Life Sciences, Swiss Federal Institute of Technology (EPFL), Geneva, Switzerland; 4 Centre d’Economie de la Sorbonne, CNRS UMR 8174 Paris, France; 5 INSERM, Cognitive Neuroimaging Unit, Gif sur Yvette, 91191 France; 6 Commissariat à l’Energie Atomique, Direction des Sciences du Vivant, I2BM, NeuroSpin center, Gif sur Yvette, 91191 France; 7 Department of Neurology, University Hospital Geneva, Geneva, Switzerland

## Abstract

In physics “entrainment” refers to the synchronization of two coupled oscillators with similar fundamental frequencies. In behavioral science, entrainment refers to the tendency of humans to synchronize their movements with rhythmic stimuli. Here, we asked whether human subjects performing a tapping task would entrain their tapping to an undetected auditory rhythm surreptitiously introduced in the guise of ambient background noise in the room. Subjects performed two different tasks, one in which they tapped their finger at a steady rate of their own choosing and one in which they performed a single abrupt finger tap on each trial after a delay of their own choosing. In both cases we found that subjects tended to tap in phase with the inducing modulation, with some variability in the preferred phase across subjects, consistent with prior research. In the repetitive tapping task, if the frequency of the inducing stimulus was far from the subject’s own self-paced frequency, then entrainment was abolished, consistent with the properties of entrainment in physics. Thus, undetected ambient noise can influence self-generated movements. This suggests that uncued decisions to act are never completely endogenous, but are subject to subtle unnoticed influences from the sensory environment.

## Introduction

Rarely is our sensory context devoid of sound, and subtle sounds in the background might influence our behavior in ways that we are not aware of. For example, imagine that you are humming a tune while in front of the stove cooking, and only later do you realize that you happened to be humming in the key suggested by the ventilation fan. Or, while writing a letter you start tapping your fingers on the table thinking of what to write next, only later realizing that you were tapping in sync with a cricket outside in the garden. We sought to experimentally examine the effect of undetected background auditory noise on *self-paced* movements (repetitive movement where the pace is self-chosen) and *self-initiated* movement (single movement initiated at a self-chosen time, without any sensory cue). We also assessed whether this effect satisfies the definition of entrainment found in physics by happening only when the two frequencies are close to each other^1^, and whether it operates in the same way for periodic movements and abrupt one-time movements.

In experiments where an ongoing stimulus is modulated or perturbed, it is important to distinguish between an above-threshold stimulus and an above-threshold perturbation (or modulation): the perturbation may be below threshold even if the stimulus itself is above threshold. Or both can be below threshold. Previous research on sensorimotor synchronization (SMS) using tapping tasks has shown that subjects make automatic corrections to their finger tapping in response to small perturbations in the timing of an above-threshold periodic auditory stimulus^2^. This has been shown even for perturbations that were well below the detection threshold^3,4^, (i.e., the phasic perturbations were below threshold, while the stimuli themselves were well above threshold). More generally, a number of prior studies have provided evidence that behavioral responses to distractor sequences (e.g. phase correction) can be automatic and involuntary^5–8^. Prior studies have also shown that human movements may become unintentionally synchronized with a plainly-audible, but irrelevant environmental rhythm^1,9,10^. A more recent study^11^ found that runners spontaneously adapt their cadence to the tempo of the music they are listening to while running.

However, in all of the above-mentioned studies the stimuli to which the subjects entrained their movements were plainly audible or visible and were an overt part of the experiment. Here we wanted to ask whether subjects would spontaneously and involuntarily entrain to a subtle periodic signal even when that signal went completely unnoticed. Entrainment under these conditions would imply that auditory processing impacts motor actions in the absence of awareness, which would contribute to the growing evidence of cross-modal unconscious processing^12,13^. In addition, we wanted to examine the extent to which such unconscious signals might influence different behaviors, by testing their impact on a simple repetitive tapping task, and on a task requiring participants to perform a single abrupt spontaneous tap on each trial after a random self-chosen delay.

**Figure 1:**
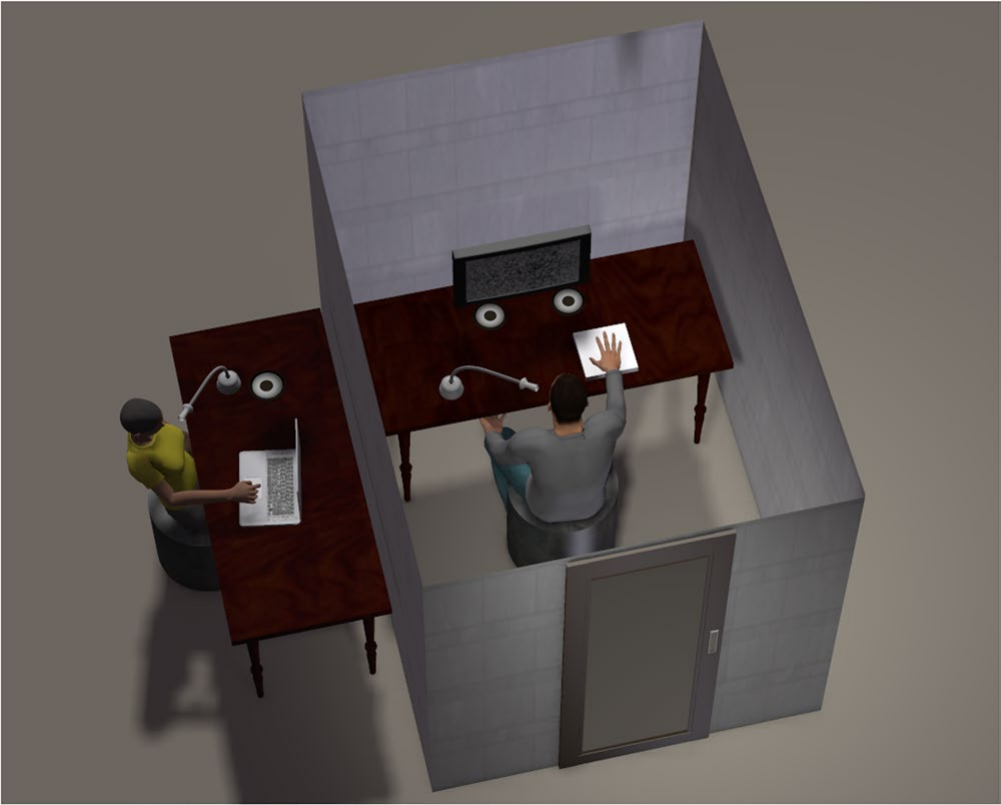
Representation of the experiment setup. The sound-proof testing chamber is shown without its roof.

In experiment 1, we had subjects perform a self-paced repetitive tapping task inside of a sound-proof testing chamber. We communicated with the subjects during the experiment via an intercom through which we surreptitiously introduced continuous faint background noise (< ~ 20 db; sound file *sound_sample.wav* in the supplementary online material). Although the noise was present continuously throughout the experiment, we could transition between periodic and non-periodic modulation of the noise (Fig. 1). Unlike in previous experiments, even the noise itself often went completely unnoticed (we confirmed this using a structured questionnaire delivered after the experiment, see Methods). In this experiment subjects exhibited statistically significant entrainment to the sine-wave modulated noise, but only when the frequency of the modulation was close to the subject’s own preferred tapping frequency, consistent with the physics of entrainment, and with prior studies^1^.

We also had subjects perform a spontaneous self-initiated movement task wherein they performed a single abrupt finger-tap spontaneously at a random moment. In this case we found that single one-time finger taps also had a significantly consistent phase relationship with the noise modulation, but unlike with repetitive tapping, this effect could also be present when the modulation frequency was not close the subject’s preferred tapping frequency. For both kinds of task we used a structured post-experiment questionnaire to evaluate whether or not each subject had been aware of the noise and found that the effects remained significant even when the analyses were restricted to subjects who had not been aware of the noise.

## Materials and Methods

### Human subjects

A total of 60 subjects participated in the study: 30 in experiment 1 (15 males, mean age = 22.0 years, SD = 3.6 years) and 30 in experiment 2 (11 males, mean age = 25.3 years, SD = 4.1 years). Data from three of the subjects in experiment 1 could not be used (N = 27 in experiment 1): one subject could not perform the task properly; one subject had a mean U-value that was an extreme outlier relative to the other subjects (absolute distance from the median was more than 2.5 times the inter-quartile range); and the data from a third subject could not be saved due to a technical problem. All subjects were right-handed, had normal hearing, and no psychiatric or neurological history. They were naive to the purpose of the study and gave informed consent, in accordance with institutional guidelines and the Declaration of Helsinki. The experimental protocol was approved by the *commission cantonale d’éthique de la recherche sur l’être humain* of the canton de Vaud, and all methods were performed in accordance with the relevant guidelines and regulations. Experiment 1 was carried out at the École Polytechnique Fédérale de Lausanne (EPFL), Department of Life Sciences, Lausanne, Switzerland. Experiment 2 was carried out at the Campus Biotech in Geneva, Switzerland.

### Stimuli and task

From the point of view of the subjects, there were no stimuli in this experiment. However, we surreptitiously introduced a small amount of noise through a pair of computer speakers placed on either side of the base of the computer display on the table in front of the subject (Fig. 1). Also on the table in front of the subject was a small microphone. The speakers and microphone were placed in the sound-proof testing room ostensibly to serve as an intercom through which we communicated with the subject from outside of the testing room without having to open the door. However, the real purpose of the speaker was to deliver auditory noise stimulation. The amplitude of the noise was adjusted so as to be barely audible (< ~20 dB) when the door to the testing room was closed and the subject was completely still, and even then hardly noticeable and easily ignored (sound file *sound_sample.wav* in the supplementary online material).

To create the noise stimuli we randomly generated spikes, each with random sign (+1 or −1), and then softened the result by imposing a $1/{f}^{\beta }$ power spectrum with *β* = 1.5 (i.e., we made the noise more pink). During the experiment we could modulate the temporal density of these spikes with a sinusoidal envelope. The magnitude of the modulation was set so as to be just barely detectable (spike probability oscillating between 0.05 and 0.1 at a sampling rate of 22050 Hz) (sound file *sound_sample.wav* in the supplementary online material). For the control condition, the modulation envelope was a random series of pulses with the intervals in between pulses drawn from a Poisson distribution (lambda = 1).

In experiment 1 (N = 27), subjects performed a simple self-paced repetitive finger-tapping task (“rhythmic task”). A subset of these subjects (N = 10) also performed (in separate blocks) a task in which they had to initiate a single abrupt tap on each trial at a spontaneously chosen moment (“single-tap task”). Tapping blocks were always done in pairs, the first of which was a rhythmic task lasting 30 seconds and the second of which was either a rhythmic task or a single-tap task lasting 60 seconds. For the subject these were just two consecutive blocks, but in fact we used the data from the first block (the “estimation” block), with steady unmodulated noise in the background, to estimate the subject’s preferred tapping rate. We then used that to determine the modulation frequency used in the second block, which could be equal to the subject’s mean tapping rate (“near” condition), 1.5 times the subject’s mean tapping rate (“far” condition), or the modulation could be aperiodic (“baseline” condition).

A new group of participants was recruited for Experiment 2 (N = 30), which was focused on the single-tap task. In this experiment blocks of trials were also done in pairs, the first of which was the 30-second estimation block, and the second of which was always the single-tap task for 60 seconds. Each trial began with the appearance of a written instruction telling the subject to tap their finger once whenever s/he wanted to. This remained on the screen for 2 seconds after which it disappeared cueing the subject that s/he can now perform the finger tap at any time. After performing the single finger tap, there was a jittered ITI of approximately 1 second and then the instruction reappeared cueing the start of the next trial.

Subjects tapped on a custom-made sensor (Salomon *et al*., 2016) consisting of a wooden tray (25 × 25 cm) with a small copper plate (width: 2.5 cm, length: 9 cm) connected to a wristband with a 2.5 × 2 cm ground electrode. The sensor was connected with an Ethernet cable to an Arduino Uno microprocessor, which was connected to the computer by a USB cable. For each tap, we recorded both the time of the finger lift and the time of the finger drop by measuring electrical conductivity between the finger and the copper plate. Subjects were instructed that their index finger should remain in contact with the copper plate by default (i.e. in the “down” position). Each finger tap was then composed of a “lift” followed immediately by a “drop” of the finger back to the default position.

At the end of the experiment, all subjects completed a structured questionnaire, which served to estimate awareness of the auditory noise stimulation. On the questionnaire we asked (1) whether the conditions in which they performed the experiment were quiet enough to perform the tasks, (2) whether they noticed any noise during the experiment, and if yes (3) whether they noticed any rhythmic component in the noise. Finally, if they noticed a rhythm in the noise, we asked (4) whether or not they thought it influenced their tapping behavior. The questions were revealed one at a time in this order, and the subject was not allowed to see the next question until s/he had finished responding to the current one.

### Data analyses and statistics

The onsets of finger drops (i.e., the time at which the finger touched the plate after an initial lift) and the continuous time-varying phase of the noise modulation were extracted to compute the phase of the noise modulation at the time of each finger tap across blocks and participants. Unintentional synchronization of movement to external stimuli is known to be somewhat unstable, with movements being attracted alternatively to either 0° or 180° rather than simply being phase locked^1,11^, and tending to drift in and out of phase^14,15^. For auditory stimuli in particular phase locking can apparently happen at a variety of different angles, not just 0° or 180°, and the angle tends to be different for different subjects^16^. Therefore we chose to use a statistical test that is sensitive to non-uniformity in the distribution of phases, rather than being sensitive to the concentration of phase angles in a single region of phase space. Each subject’s phase values were submitted to Rao’s spacing test of uniformity (Rao, 1976), providing U-values as a non-uniformity index for the distribution of phases. U-values greater or smaller than the median by more than 2.5 times the interquartile range were excluded, and the remaining U-values were averaged across blocks. The significance of a main effect of condition was assessed using linear mixed effects models, with U-value ranks averaged across blocks as the dependent variable^17^, condition as a fixed effect, and intercepts for subjects as random effects. Note that condition could not be treated as a random effect, otherwise the number of observations would be smaller than the number of random effects. In all models, p-values were obtained by likelihood ratio tests, and degrees of freedom were estimated using the Satterthwaite approximation. Statistical significance of the difference in U-values between conditions was assessed using permutation tests: a null distribution of the mean difference in U-values at the group-level was created by shuffling the condition labels over 5000 iterations. In line with 2-sided tests, p-values were estimated by counting the proportion of shuffled samples exceeding the observed average difference in U-values in the near (or far) versus baseline conditions. All analyses were performed with R (2016) using the lme4 and lmerTest packages (Bates, Maechler, Bolker, & Walker, 2014; Kuznetsova, Brockhoff, & Christensen, 2014).

### Monte Carlo Simulation

If two oscillators have the same frequency, then they will have a consistent phase relationship over time – no entrainment necessary^1,10^. Even if their frequencies are not perfectly constant over time, as long as the variance of the interval distribution is very small relative to the window of time over which the oscillators are sampled, then there will be a relatively consistent phase relationship. Thus if a subject were to tap at a constant rate throughout the training block, and then maintain exactly the same rate of tapping throughout the subsequent “near” block, then there would be a consistent phase relationship without necessarily implying entrainment. In order to control for this possibility we performed a Monte Carlo simulation in which, for each “near” block of each subject, we drew random intervals from a distribution with the same mean and variance as this particular block. We used the mean and variance to generate 1000 surrogate sequences of intervals and then computed the 1000 corresponding U-values on the phases of the inducing stimulus at the time of each surrogate tap. If the subject’s mean frequency was close enough to that of the inducing stimulus and the variance of the subject’s intervals was small enough, then the surrogate taps should yield U-values close to what was actually observed.

## Results

**Figure 2:**
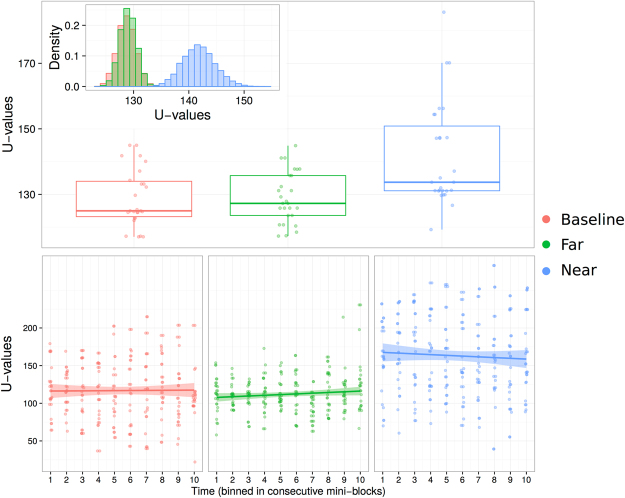
Results of experiment 1. Upper panel shows a box plot of the results in the three conditions, baseline (red), far (green), near (blue). Inset shows the result of the permutation test. Lower panel shows the same results as the upper panel, but separately for each mini-block (one dot = one subject).

On average, participants tapped at a frequency of 1.44 Hz (+/− 0.12 Hz). A linear mixed model on ranked U-values revealed a main effect of condition (F(2,77) = 11.52, p < 0.001), showing that U-values were smaller in the baseline condition (mean U-value = 128.77 + /− 8.78) compared to the near (mean U-value = 141.85 +/− 15.83; permutation test: p < 0.001), but not the far condition (mean U-value = 129.02 + /− 7.81; permutation test: p = 0.47) (Fig. 2). In the near condition 8 of the 27 subjects had U-values that were individually significant at p < 0.05 (p < 0.0001, binomial test).

The average phase values across subjects did not depart from uniformity (Rao’s uniformity test, p > 0.1), indicating that subjects were phase locked at different angles. However, as mentioned in the Methods section, unintentional synchronization of movement to auditory stimuli is known to be somewhat unstable, with movements being attracted to different phase angles depending on the subject, rather than simply being phase locked^16^.

To test whether entrainment in the near condition changed over time during the course of each trial, we computed U-values within 10 contiguous mini-blocks each containing 10% of the total number of taps in a trial. A linear mixed model on ranked U-values with intercepts for subjects as random effects, and a per-subject random slope for the effect of condition and mini-blocks confirmed the main effect of condition reported above (F(2,50.36) = 20.19, p < 0.001), but did not reveal any effect of mini-block (F(1,25.40) = 0.08, p = 0.79) nor an interaction between mini-block and condition (F(2,690.90 = 1.74, p = 0.18) (Fig. 2). On the structured questionnaire following the experiment, no subject reported perceiving noises or noise modulations, suggesting that entrainment in the near condition occurred despite unawareness.

Taken together, these results suggest that entrainment does occur for self-paced tapping in the absence of awareness. Consistent with what is found in physics and in prior studies^1^, such entrainment was observed only when the entraining frequency was close to the subject’s preferred pace.

In order to control for the possibility that the apparent entrainment may have resulted simply because subjects maintained a very precise and constant rate of tapping between the “training” and “near” blocks, we performed a Monte Carlo simulation (see Methods). If the subject’s mean frequency was close enough to that of the inducing stimulus and the variance of the subject’s intervals was small enough, then the surrogate data produced by the simulation should have yielded a significant value for Rao’s U. The difference in U-values between the near and baseline condition over 1000 Monte Carlo simulations was 7.31 (SD = 2.31), significantly different from 0 (Monte Carlo p-value < 0.001). This suggests that a part of the observed effect could have stemmed from a consistent tapping frequency between the training and the near condition. However, the average difference in U-values between the near and baseline conditions observed in all three experiments was 13.02 (SD = 18.42), superior to 99.1% of the Monte Carlo differences. This suggests that the observed effect is not completely accounted for by a consistent frequency of tapping, and that actual entrainment did occur in the near condition.

A subset of, subjects also had to perform a spontaneous self-initiated movement task, wherein they perform a single spontaneous tap on each trial after a random delay of their own choosing. A linear mixed model on the ranked U-values in these trials revealed a trend for a main effect of condition (F(2,24) = 3.22, p = 0.06). We further explored this main effect, and found that the U-values were significantly smaller in the baseline condition (mean U-value = 121.12 + /− 5.21) compared to both the near (mean U-value = 130.96 + /− 5.90; permutation test: p = 0.02) and to the far condition (mean U-value = 130.00 + /− 7.23; permutation test: p = 0.004). As opposed to what we found previously, these results suggest that the noise modulation in both the “near” and “far” conditions had an effect on the finger tap initiation.

A replication of the above preliminary result was obtained in Experiment 2, which included 30 participants. The linear mixed model on the ranked U-values in these trials revealed a significant main effect of condition (F(2,60) = 6.41, p = 0.003), with smaller U-values in the baseline condition (mean U-value = 123.98 + /− 3.77) compared to both the near (mean U-value = 132.22 + /− 3.71; permutation test: p = 0.002) and to the far condition (mean U-value = 131.25 + /− 3.27; permutation test: p = 0.008). Compared to the previous experiments, a greater number of participants noticed a sound while they were performing the task (n = 18), and a subset of them even noticed a rhythmic pattern in it (n = 12). This may be due to the fact that Experiment 2 was performed in a different lab, with differences in terms of acoustics that we could not account for.

**Figure 3:**
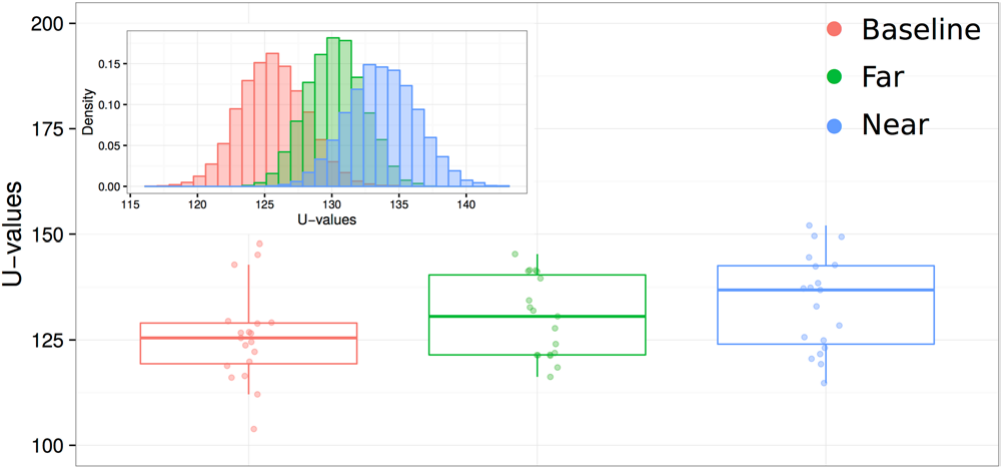
Results from experiment 2, with subjects who noticed the modulation excluded. Inset shows the results of the permutation test.

When excluding these participants, keeping only those who reported hearing no modulation, the difference in U-values between the baseline (mean U-value = 125.61 + /− 4.90) and the near condition remained significant (mean U-value = 133.79 + /− 5.12; permutation test: p = 0.008), and a trend was found for the difference between the baseline and the far condition (mean U-value = 130.22 + /− 4.25; permutation test: p = 0.08) (Fig. 3). While a significant number of subjects had individually significant U-values in experiment 1, the same was not true of experiment 2 (3 subjects out of 30 individually significant at p < 0.05), where the effect was only detectable in the aggregate across subjects. This may have been because there were far fewer taps per subject in experiment 2, due to the nature of the single-tap task.

## Discussion

At any given moment if you remain very still and listen carefully, you will likely hear all manner of background sounds – birds chirping outside, the hum of your desktop computer cooling fan, the ticking of a clock on the wall, the sound of a refrigerator, etc. These kinds of background sounds may typically go unnoticed, especially if they are relatively faint. We asked whether or not undetected rhythmic regularities in auditory background noise could influence self-paced or self-initiated movement. In order to test this, we introduced faint background noise into an intercom system and then modulated the noise in different ways in order to measure its effect on behavior in two different types of tapping tasks, under three different conditions. In one task subjects simply tapped rhythmically at their own preferred pace (task 1), and in the other task (task 2) subjects performed a single spontaneous tap after waiting an unspecified amount of time. For each task the noise could be modulated at the subject’s own preferred tapping rate (“near” condition), at 1.5 times the subject’s preferred tapping rate (“far” condition), or the modulation could be random and aperiodic (“baseline” condition). For task 1, subjects’ tapping was entrained in the “near” condition but was not entrained in the “far” or “baseline” conditions. For task 2, subjects tended to perform their single tap (one per trial) in synchrony with the modulation in both the “near” and “far” conditions. Together, these results indicate that unconscious auditory processing can impact rhythmic motor actions, and can even modulate the onset of spontaneous actions unbeknownst to the participants. This attests to the complexity of sensorimotor processing occurring in the absence of awareness (Deroy *et al*., 2016; Faivre *et al*., 2017).

Prior studies of synchronized finger tapping have demonstrated effects of undetected timing variability in otherwise supra-threshold rhythmic auditory stimuli^7,18–20^. Here we extended these findings by showing that undetected auditory regularities (rhythmic modulation of faint background noise) could influence self-paced finger tapping, even when both the inducing stimulus and its effect on behavior went completely undetected. Indeed, in experiment 1 subjects denied perceiving any sounds or sound modulations, and in experiment 2 the results remained significant even when restricted to subjects who denied perceiving any sounds or modulations. This suggests that effects in task 1 and task 2 occurred despite the absence of auditory awareness. Here, we probed auditory awareness using subjective measures at the end of experiment, since asking subjects to perform an objective task on the sound during the experiment would uncover its presence. As the sound modulations became faintly audible when attending to them, the use of objective measures was incompatible with our experimental design. Sub-threshold perturbations of supra-threshold and plainly-heard distractor sequences are known to engage corrective mechanisms automatically, without subjects intending to make corrections or even noticing that any correction had been made^20,21^. Our data suggest that such corrective mechanisms may still be engaged when the distractor sequence (in our case the background noise) goes undetected.

Entrainment not only applies to bodily movement, but to brain activity as well, and prior research has shown that entrainment of low-frequency brain oscillations might mediate the effects of expectation on reaction time^22^. This suggests a mechanism by which the auditory regularities in the present study might influence the timing of self-paced and spontaneous tapping. Recent work on uncued “self-initiated” movements^23,24^ argues that, in the absence of an imperative stimulus, the precise onset time of self-initiated movements may at least in part be determined by ongoing endogenous fluctuations of brain activity. The current work connects to those prior findings by showing that the precise onset time of uncued movements may also be influenced by exogenous fluctuations in the sensorium (a slight shift in the phase of a finger tap is equivalent to a change in the precise onset time of that movement). Future research should look at the relationship between the phase of slow EEG fluctuations and tapping times in our task.

Counter to intuition, it has previously been shown that irrelevant stimuli may have a stronger effect on behavior when they are just below threshold compared to just above^5,25^. Presumably this is because cognitive control mechanisms are only engaged when task irrelevant stimuli are processed consciously, as if cognitive control mechanisms are recruited in order to suppress distracting stimuli. Future research could test this hypothesis by examining the effect of periodic stimulation at just above and just below the perceptual threshold, and also examine how exogenous and endogenous factors interact to fix the precise onset time of movement when there is no imperative stimulus.

## Electronic supplementary material

Supplementary sound sample
